# Physically mixed graphene aerogel:carbon black (GA:CB)-supported Pt or hydrothermally synthesized GA-Pt/CB hybrid electrocatalysts for PEM fuel cells

**DOI:** 10.55730/1300-0527.3800

**Published:** 2026-05-08

**Authors:** Emine TEKE ÖNER, Melis Irmak AYDIN, Merve Nida DURSUNOĞLU, Ayşe BAYRAKÇEKEN

**Affiliations:** 1 Department of Chemical Engineering, Faculty of Engineering, Atatürk University, Erzurum, Turkiye; 2 Department of Nanoscience and Nanoengineering, Institute of Science, Atatürk University, Erzurum, Turkiye

**Keywords:** Graphene aerogel, catalyst support, hydrothermal, proton exchange membrane fuel cell

## Abstract

Graphene aerogels (GAs), having the superior properties of graphene and aerogel structures, are promising support materials for Pt nanoparticles to be used as electrocatalysts for PEM fuel cells. In this study, two routes were followed to synthesize GA-based electrocatalysts. In the first route, after GA synthesis it was physically mixed with carbon black (CB) by changing the mass ratios from 0% to 100% with 25% increments and then decorated with Pt nanoparticles via microwave irradiation, resulting in Pt/GA:CB electrocatalysts. In the second route, firstly, Pt/CB electrocatalyst was synthesized and then GA was hydrothermally added to the Pt/CB by changing the mass ratios of GA:Pt/CB from 0% to 100% with 25% increments, resulting in GA-Pt/CB electrocatalysts. The electrocatalysts synthesized were physically characterized (ICP-OES, XRD, Raman, SEM, TEM, surface area, and contact angle) and then in situ PEM fuel cell tests were performed. Following different synthesis routes resulted in different physical properties. The hydrothermally synthesized electrocatalysts gave a better PEM fuel cell performance when compared to the physically mixed ones.

## 1. Introduction

Proton exchange membrane fuel cells (PEMFCs) are promising energy conversion systems due to their lower operating temperature and higher power densities for portable and mobile applications [1]. The improvement of critical materials is of great importance for the widespread utilization of PEM fuel cells, which can compete with the existing energy systems such as internal combustion engines, batteries, and supercapacitors. Among the other fuel cell materials, electrocatalysts are crucial for a better performing fuel cell. PEM fuel cell electrocatalysts are mostly composed of carbon-supported metallic nanoparticles in which carbon black (CB), activated carbon, carbon aerogel, and graphene-based carbon structures are used as support materials [2,3].

In recent years, graphene-based nanomaterials have attracted considerable attention as PEM fuel cell catalyst support materials, particularly for the oxygen reduction reaction (ORR), which is sluggish and has to be accelerated [4]. Chemically synthesized graphene possesses surface functional groups (carbonyls, epoxides, hydroxyls, etc.) and contains lattice defects in the interlayer structure (voids and holes). These functionalities and lattice defects can stabilize and immobilize metal nanoparticles through strong metal–support interactions. Graphene structures have been designed in different ways to achieve high performance in fuel cells.

Graphene possesses excellent electrical and thermal conductivity, high mechanical strength, and high theoretical surface area [5]. In particular, 3D graphene aerogels (GAs) are a fascinating class of graphene-based materials that combine the favorable properties of graphene with those of high porosity and low-density aerogels [6]. In this context, highly conductive mesoporous GAs are potential candidates as support materials for ORR catalysts. Their electrochemical activity has been a topic of interest in recent studies. Despite these advantages, plain GA structures may still exhibit limitations in terms of mechanical stability, electrical conductivity, and processability in membrane electrode assembly (MEA) fabrication. Therefore, hybrid catalyst systems integrating GAs with conventional carbon supports have been proposed to exploit synergistic effects. In such hybrid systems, CB can provide good electrical conductivity and processability, while GAs contribute enhanced porosity and mass transport pathways, resulting in improved overall catalyst layer performance [7].

GAs can also be used in composite form for electrochemical applications. A 3D GA structure was synthesized and then composites with polypyrrole (PPy) were prepared. Furthermore, hydrophobic polydimethylsiloxane (PDMS) polymer was added to the catalyst ink to increase the hydrophobicity and durability of the GA- and PPy-GA-supported Pt catalysts. The addition of PDMS to the catalyst ink increased the electrocatalytic activity and durability of the GA-supported Pt catalyst, which was determined with cyclic voltammetry (CV) experiments [8]. Pt/GA electrocatalysts were synthesized at different pyrolysis temperatures using supercritical carbon dioxide deposition and were also tested with CV and rotating disk electrode (RDE) measurements [9]. Nitrogen-functionalized graphene materials have been studied as a promising electrocatalyst for ORR due to their catalytic activity and excellent stability in alkaline electrolytes. Co-N-functionalized GA was used as ORR catalyst in acidic electrolytes. An electrocatalyst providing significant catalytic activity and rapid mass transfer, as well as exceptional stability in acidic environments, comparable to commercial Pt/CB is presented [10].

Physically mixed graphene-based materials with different carbon structures are used as support materials for PEM fuel cell electrocatalysts. Physically mixed graphene oxide (GO) and CB hybrids in varying ratios (from 0% to 100% in 25% increments) were used to impregnate Pt to prepare a Pt/rGO/CB hybrid electrocatalyst, which resulted in a maximum power density with a low amount of CB content (25%) in a fuel cell environment [11]. Reduced electrochemically exfoliated graphene oxide (rEGO) and CB were physically mixed in water and then Pt nanoparticles were decorated with the polyol method. This hybrid structure provided high durability, very good stability, and efficient energy production with 1.8 times better performance in the fuel cell compared to the known Pt/CB electrocatalyst [12].

In another study, graphene nanoplatelets (GNPs) and CB were physically mixed in various ratios (GNPs:CB ratios from 100:0 to 50:50 in 10% increments) and Pt nanoparticles were loaded onto the hybrid supports using supercritical carbon dioxide deposition. The best fuel cell performance was obtained for the support having a GNPs:CB mass ratio of 60:40 [13]. A hybrid support structure consisting of carbon nanotubes and reduced graphene oxide (rGO) was used for the decoration of Pt nanoparticles via microwave irradiation. The mixing of rGO with CNT was achieved by two different routes. In the first route, materials were mixed simultaneously; in the second route, firstly CNT was mixed and then rGO was added. The supports prepared with the second route gave better fuel cell performance when compared to the first route [14]. Pt decorated physically mixed CB and rGO [15] and physically mixed N-doped CB and graphene [16] support materials were used to improve the electrocatalytic activity in fuel cells. High electrochemical surface area, better performance for ORR, high power density in PEMFC tests, and long-term stability were obtained.

Another route for obtaining hybrid electrocatalysts by using different carbon structures is firstly obtaining a carbon-supported Pt electrocatalyst and making composites of these electrocatalysts with other carbon-based materials. Physically mixed Pt/G catalyst with CB mass ratios ranging from 0% to 50% was used as cathode catalyst. It was observed that 25% CB in the hybrid catalyst gave the best performance [17]. Physical mixing of Pt-boron-doped graphene with 0%–40% CB in 10% increments was investigated. It was shown that the catalyst containing 30% CB had the best electrochemical surface area for fuel cell performance, but further increase resulted in decreased performance [18]. In another study, the Pt/rGO catalyst was physically mixed with CB at mass ratios of 1:1 and 2:1. The authors observed that the addition of CB to the Pt/rGO catalyst increased the ORR activity and stability. A slight difference was observed during the change in the mass ratio of catalyst to CB, with the 1:1 ratio providing better performance [19].

The enhancement in electrochemical activity in GA-based hybrid catalysts is not only attributed to increased surface area but rather arises from multiple synergistic mechanisms such as:

- Improving electron transport across the catalyst layer via formation of a 3D conductive network [20]
- Mitigating flooding issues under high current densities due to hierarchical pore structure, which resulted in enhancement in mass transport of oxygen and water removal with high hydrophobicity [21]
- Promoting stronger metal–support interactions with defect sites and functional groups in GAs, which improves Pt dispersion and stability [22].

The synthesis route plays a critical role in determining the interfacial properties between GA and Pt/CB which directly influences catalyst performance. In particular, different synthesis approaches, such as physical mixing and hydrothermal integration, can lead to significantly different structural and electrochemical properties. Physical mixing offers simplicity but often results in weak interfacial interactions and nonuniform distribution of components. In contrast, hydrothermal synthesis enables better integration of GA with Pt/CB, promoting stronger interfacial bonding, improved dispersion of catalytic nanoparticles, and more efficient utilization of active sites [22].

Graphene is a 2D material, whereas GA is a 3D material. In the literature, most of the studies are carried out with graphene, GO, or rGO as support materials. There are few studies in the literature that cover the utilization of GA as support material for PEM fuel cell electrocatalysts and only ex situ tests were performed in these studies [8,9]; mostly GAs are used as direct liquid fuel cell electrocatalyst support materials [7,23]. GA is a promising material for the utilization as an electrocatalyst support material in PEM fuel cells due to its outstanding structural properties; however, its electrocatalytic activity requires further investigation and clarification in terms of in situ PEM fuel cell tests.

Literature studies showed that hybrid electrocatalysts resulted in superior fuel cell performance. Moreover, following different routes for the preparation of hybrid PEM fuel cell electrocatalysts significantly affects the performance of PEM fuel cells. In the present study, a promising support material, GA, was synthesized and used as a hybrid material. It was aimed to prepare hybrid PEM fuel cell electrocatalysts following two different routes including physical mixing of CB with GA as support material for Pt nanoparticles and hydrothermal synthesis of GA-Pt/CB electrocatalyst. Corresponding physical characterizations and in situ PEM fuel cell tests were performed.

## 2. Experimental

Two routes were followed in order to synthesize the corresponding electrocatalysts.

### 2.1. Physically mixed graphene aerogel/carbon black (GA/CB)-supported Pt electrocatalyst synthesis

In the first route, firstly GA was synthesized and then CB composites were obtained. After the preparation of these support materials, Pt was decorated over these supports via microwave irradiation.

### 2.2. GA synthesis

GO, required for the synthesis of the 3D GA structure, was obtained from commercial graphite powder using the modified Hummer method [24,25]. In the present study, graphite (Alfa Aesar) was subjected to preoxidation. This preoxidation process was performed to increase the oxidation strength of the Hummer method and to open the gap between the graphene layers. Twenty grams of graphite, 50 mL of H_2_SO_4_, 10 g of K_2_S_2_O_8_, and 10 g of P_2_O_5_ were put in a beaker and kept at 80 °C for 6 h for preoxidation. The resulting mixture was washed with distilled water, dried at room temperature, and then used in the Hummer method. Three grams of graphite oxide, 69 mL of H_2_SO_4_, and 1.5 g of NaNO_3_ were mixed in a beaker. The mixture was placed in an ice bath and 9 g of KMnO_4_ was slowly added, ensuring the temperature did not exceed 5 °C. The mixture was stirred in the ice bath for 30 min and then removed from it. The temperature was brought to 35 °C and stirred for another 3 h. Then 138 mL of pure water was added very slowly to the mixture and the temperature was maintained at around 50 °C. The ice bath was used for easier temperature control at this stage. After stirring for 30 min, an additional 420 mL of water was added to the solution. Next, 15 mL of 30% H_2_O_2_ was added dropwise. After this addition, the mixture turned light brown. This color change indicates that the GO synthesis steps were progressing correctly. At this stage, the temperature was maintained at around 40 °C. The resulting mixture was washed with 250 mL of 10% HCl solution. After this process, it was washed twice with pure water and the mixture was dried in the oven at 60 °C. GA synthesis was carried out by freeze-drying the resulting hydrogel product after its synthesis. Then 200 mg of graphene oxide was dissolved in 100 mL of distilled water. This mixture was kept in a Teflon-lined autoclave at 180 °C for 12 h. The resulting hydrogel was kept in the freezer overnight and placed in a lyophilizer at −80 °C for 24 h and then GA was obtained. After GA synthesis, GA/CB composites were prepared by physically mixing the synthesized GA with CB (Vulcan XC72R) by changing the mass ratios from 25% to 75%. Pt nanoparticles were decorated on these support materials via microwave irradiation [8].

### 2.3. Hydrothermal synthesis of GA-Pt/CB electrocatalysts

GO was synthesized using the Hummer method to prepare GA-Pt/CB electrocatalysts. In this route, firstly Pt/CB catalyst was synthesized using microwave irradiation, and the electrocatalysts were prepared by changing the mass ratios of GO and Pt/CB amounts (25%, 50%, and 75%) during the hydrothermal synthesis. First, synthesized GO was dissolved in 100 mL of distilled water. Then the determined amount of Pt/CB for the corresponding mass ratio was added to 100 mL of GO aqueous solution. The mixture was then autoclaved and incubated at 180 °C for 12 h hydrothermally. The resulting hydrogel structures were freeze-dried, resulting in the GA-Pt/CB hydrothermally synthesized electrocatalysts [26].

### 2.4. Synthesis of electrocatalysts via microwave irradiation

Pt-based electrocatalysts for both synthesis routes were prepared using microwave irradiation. GA/CB for the first route or CB for the second route were mixed with H_2_PtCl_6_ (2.63 mL) and ethylene glycol (50 mL). The mixture was first stirred in a homogenizer for 5 min and then stirred in a magnetic stirrer for 1 h. Next, the mixture was kept for 1 min in a domestic microwave oven at 800 W power for the reduction process. The mixture was then placed in an ice bath and, after cooling, centrifuged three times with acetone and distilled water to remove impurities. The prepared materials were dried in an oven at 100 °C for 12 h [8,27]. The nominal Pt percentage targeted was 20% by mass.

### 2.5. Physical characterization of the electrocatalysts

Qualitative and quantitative analysis of the elemental composition of the synthesized electrocatalysts was performed using an Agilent 7800 brand ICP-OES instrument. The crystal structures of the synthesized materials were determined with a PANalytical Empyrean brand XRD device in the range of 10–90° (2ϴ) with a Cu Kα (λ = 1.54 Å) radiation source. Raman analyses of the synthesized support and electrocatalysts were performed using a WITech alpha 300R instrument. The surface morphology and structure of the synthesized materials were analyzed using a Zeiss Sigma 300 device. TEM analyses of the synthesized electrocatalysts were performed using a Hitachi HighTech HT7700. BET analysis of the synthesized support materials was performed with a Micromeritics 3Flex device. The static contact angle measurements were performed using an Attension Theta Flex Contact Angle instrument.

### 2.6. PEM fuel cell tests

The catalyst ink solution was obtained by mixing the corresponding electrocatalyst, Nafion solution, 2-propanol, and water in specific proportions. The solution was stirred in an ultrasonic bath for 30 min to ensure homogeneity. The catalyst solution prepared was applied to the gas diffusion layer (GDL, GDL 34BC, Sigracet) on a heated vacuum plate using the traditional brushing method. When preparing the catalyst ink, the quantities of the materials to be used were theoretically calculated based on the amount of Pt to be coated and the amount targeted was 0.4 mgPt/cm^2^. After preparing the gas diffusion electrodes (GDEs) for the anode and cathode sides, a Nafion 212 membrane was placed between these two GDEs and hot-pressed for 3 min at 130 °C and 400 psi and MEA was obtained. Fuel cell polarization curves of the resulting MEA structures sandwiched in between the single fuel cell hardware were obtained using a Henatech single fuel cell test station. Measurements were taken at 70 °C for cell temperature and anode and cathode humidification temperatures. Nitrogen gas was fed into the system until the system reached this temperature, and temperatures were allowed to equilibrate. After the system heated, hydrogen gas was fed into the system at the anode and oxygen gas at the cathode at a flow rate of 0.25 slpm. The system was operated at 0.1 V until temperatures reached equilibrium. Once the system reached equilibrium, performance measurements were taken at every 30 min [28]. In the present study, other than ex situ CV and RDE tests, in situ PEM fuel cell testing was carried out in order to highlight the electrocatalytic activity of the electrocatalysts in real time conditions [29–31].

## 3. Results and discussion

### 3.1. Physical characterization results

Pt nanoparticles were decorated onto the synthesized support materials using microwave irradiation. Table 1 shows the Pt mass percentages of the electrocatalysts obtained from ICP-MS analysis. Hydrothermally synthesized electrocatalysts were decorated with less Pt when compared to the physically mixed GA:CB-supported Pt electrocatalysts. However, for both synthesis routes the Pt loadings were lower than the nominal 20 wt.% value [32]. Addition of Pt/CB electrocatalyst during the hydrothermal synthesis of GA from GO at different mass ratios resulted in lower Pt amounts due to the increase in the support amount in the synthesis environment.

**Figure 1:**
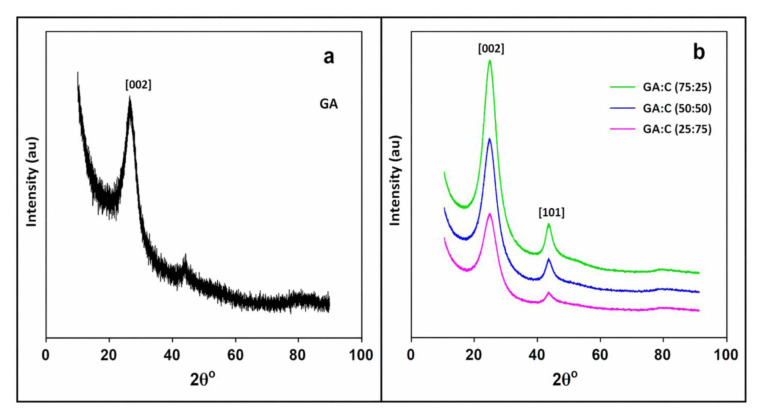
XRD analysis of (a) GA and (b) GA:CB support materials.

The XRD analysis results of GA and physically mixed GA:CB composites are given in Figure 1. The sharp diffraction peak observed at 25.6° in Figure 1a corresponds to the (002) plane and is consistent with the diffraction peaks of the GA structure in the literature [33]. Figure 1b shows the XRD analysis results of GA:CB support materials and two diffraction peaks attributed to graphitic carbon structures. The diffraction peak at 24.30° corresponds to the (002) plane, while the diffraction peak at 43.36° corresponds to the (101) plane [34–36].

**Figure 2:**
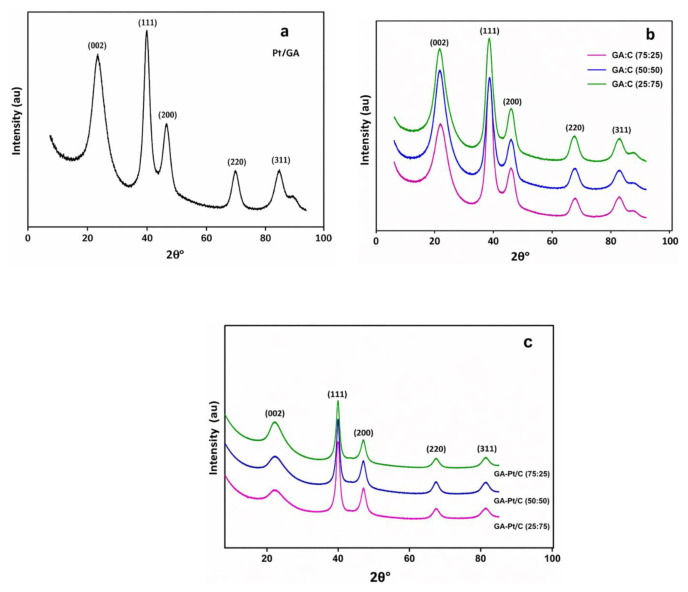
XRD analysis of (a) Pt/GA, (b) physically mixed GA:CB-supported Pt electrocatalysts, (c) hydrothermally synthesized GA-Pt/CB electrocatalysts.

The XRD results of the GA and GA:CB-supported Pt electrocatalysts and also hydrothermally synthesized electrocatalysts are given in Figure 2. The diffraction peak observed at 24.50° for the synthesized catalysts is attributed to the (002) plane of carbon [37]. The diffraction peaks at 2ϴ = 39.90°, 46.10°, 67.70°, and 81.67° observed in the electrocatalysts are the peaks belonging to the (111), (200), (220), and (311) planes of the face-centered cubic (fcc) crystal Pt structure for both synthesis routes, respectively [11,38,39].

**Figure 3:**
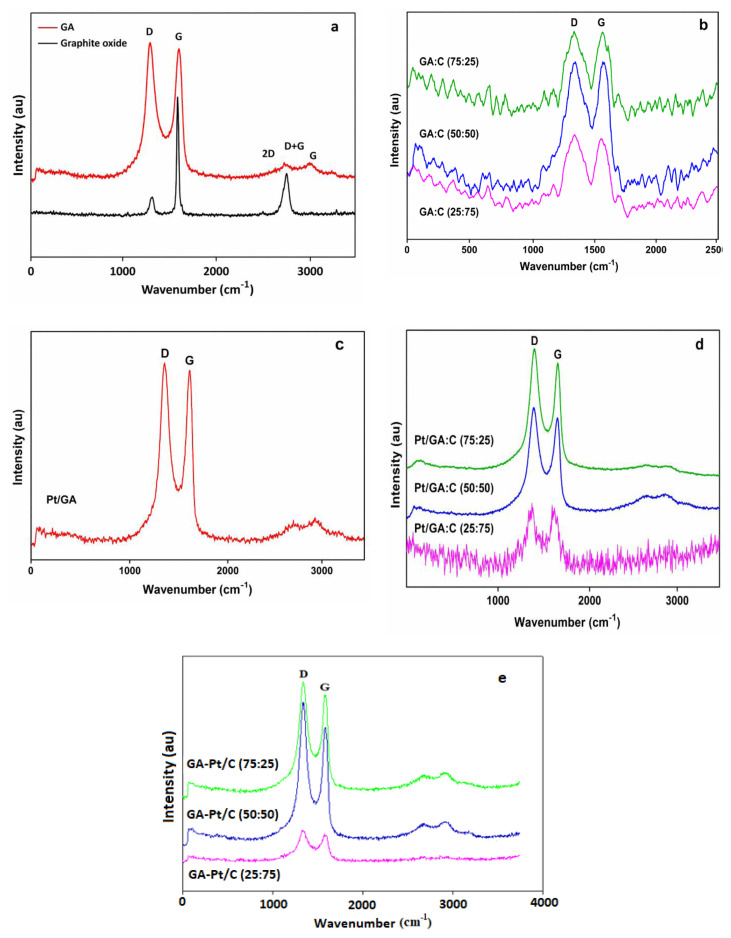
Raman spectra of (a) GA and graphite oxide, (b) physically mixed GA:CB supports, (c) Pt/GA, (d) physically mixed GA:CB-supported Pt electrocatalysts, (e) hydrothermally synthesized GA-Pt/CB electrocatalysts.

Raman measurements were used to analyze the structural changes of the synthesized support materials and electrocatalysts. The data obtained from the analysis are plotted in Figure 3. The Raman spectra of GA and physically mixed GA:CB hybrids are given in Figures 3a and 3b. Two Raman peaks centered at 1336 cm^−1^ and 1590 cm^−1^ are seen in the figures, and these peaks are attributed to the D and G bands [40]. In Raman spectroscopy, the D band originates from structural defects or imperfections found in graphite-based materials, which is formed by the out-of-plane motion of atoms resulting from sp^3^ hybridization. The G band is attributed to sp^2^ hybridized ordered graphitic carbon, which is formed by the linear inward and outward motion of sp^2^ hybrid atoms. The D/G band intensity ratio (ID/IG) is generally used to measure the degree of structural disorder, and it is thought that a higher ratio indicates greater structural disorder. The ID/IG ratios for GA and GA:CB hybrids were 0.843. Carbon addition to the GA did not change this ratio. The ID/IG ratio of GO was 0.860. The Raman spectra for Pt/GA and physically mixed GA:CB-supported Pt electrocatalysts are given in Figures 3c and 3d. Two Raman peaks centered at 1362 cm^−1^ and 1582 cm^−1^ are observed for each sample [9] with an ID/IG ratio of 0.860. For the hybrid electrocatalysts the D and G bands are seen with peaks centered at approximately 1340 cm^−1^ and 1590 cm^−1^ and the ID/IG ratio was 0.847. The addition of Pt increased the defect sites in the structure. Raman analyses of hydrothermally synthesized GA-Pt/CB hybrid electrocatalysts are given in Figure 3e. Two peaks (D and G bands) centered at 1337–1343 cm^−1^ and 1582–1591 cm^−1^ are observed for the electrocatalysts synthesized. In the literature, 2D, D+G, and 2G peaks are also mentioned in some samples. The observation of the 2D band is attributed to the presence of several-layer graphene in the structure. The ID/IG ratios were 0.860 for GA-Pt/CB (75:25), 0.85 for GA-Pt/CB (50:50), and 0.840 for GA-Pt/CB (25:75). It was observed that the ID/IG ratio increased as the amount of GA increased in the structure.

**Figure 4:**
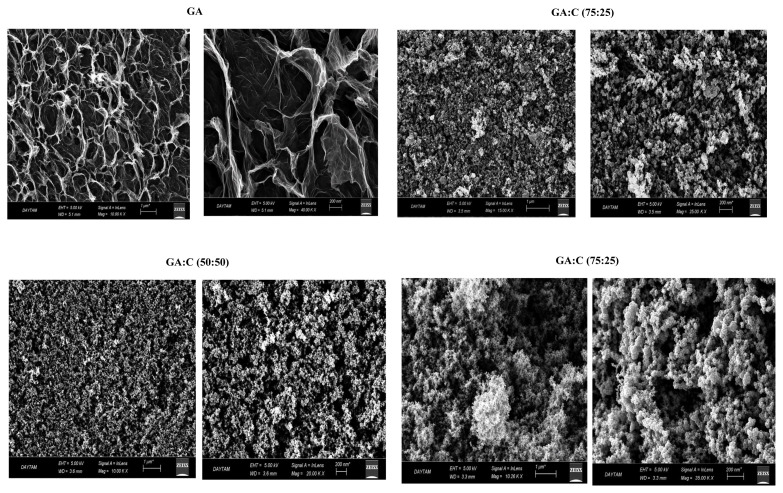
SEM images of GA and physically mixed GA:CB support materials.

SEM analysis was performed to examine the morphological structure of GA and physically mixed support materials. Figure 4 shows SEM images of GA and its hybrid materials at 1 μm and 200 nm magnifications. The SEM images of GA clearly reveal the 3D structure of the material [41]. The images of the hybrid materials show the successful dispersion of CB between the GA layers.

**Figure 5:**
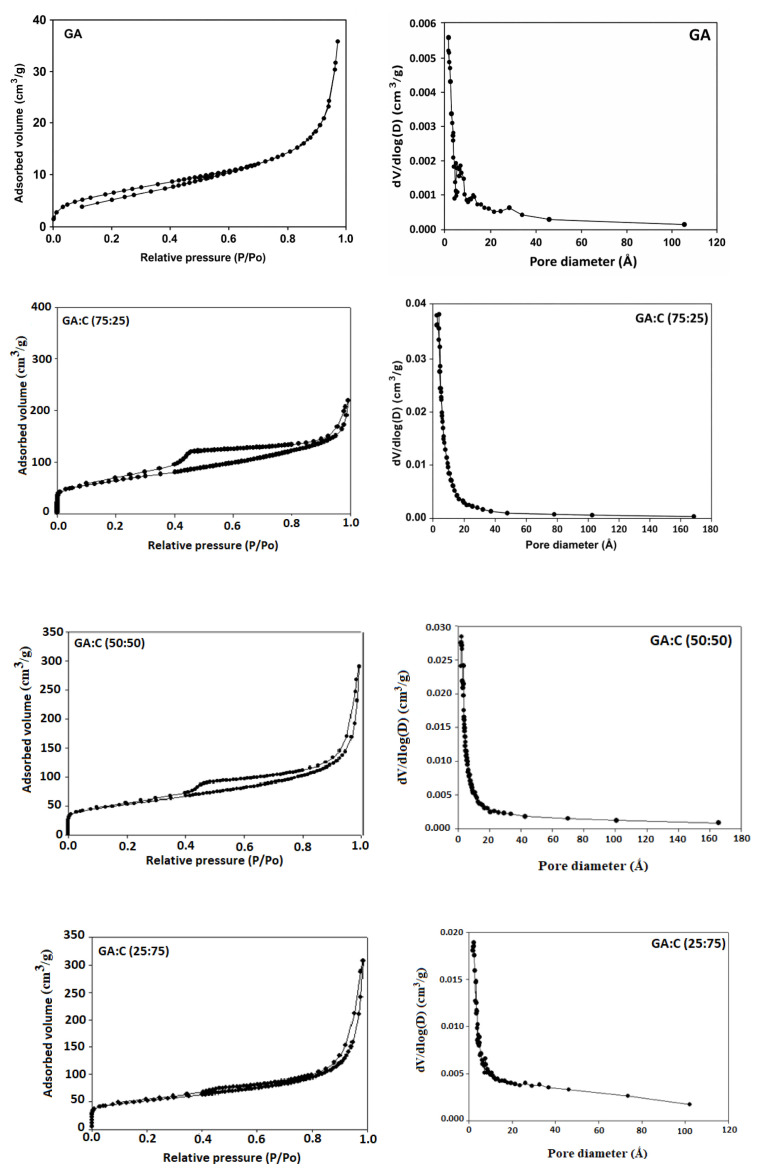
Nitrogen adsorption/desorption isotherms and pore size distributions of GA and physically mixed GA:CB support materials.

Nitrogen adsorption/desorption isotherms and pore size distributions of the GA and GA:CB support materials are shown in Figure 5. The isotherm of the GA and GA:CB support materials is type IV, which reflects the mesoporous structure. The hysteresis loop of all materials is H3. The H3 hysteresis loop is associated with the slit-shaped pores between the parallel layers [42]. The data obtained from the BET analysis are presented in Table 2. The BET surface areas range from 265.3 to 178.6 m^2^/g. The GA support material exhibited the highest surface area, and the surface area decreased as the GA content decreased in the composites.

**Figure 6:**
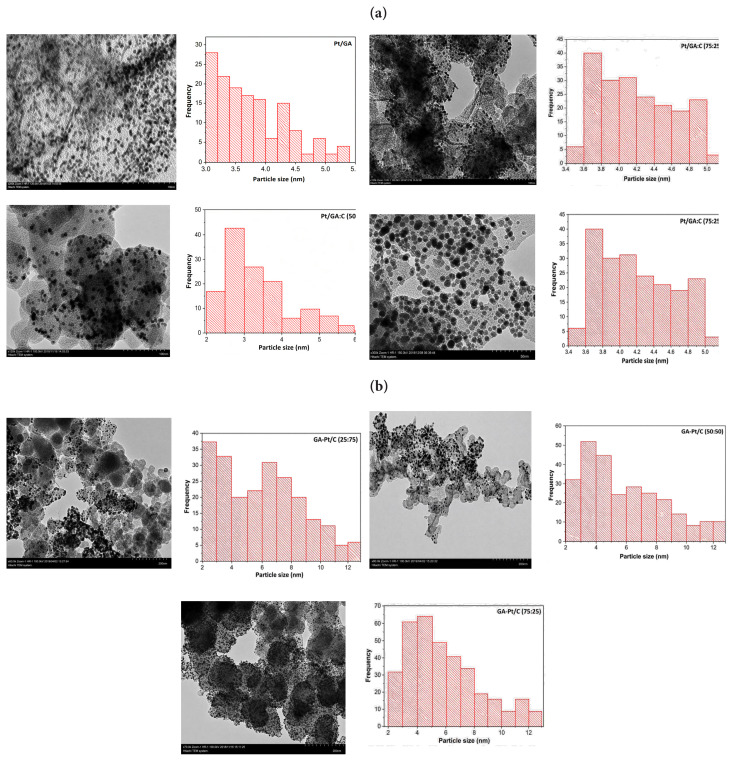
TEM images and the particle size histograms of the electrocatalysts a) GA and physically mixed GA:CB-supported Pt electrocatalysts, b) hydrothermally synthesized electrocatalysts.

The TEM images are used to evaluate the distribution, homogeneity, and particle size of the Pt nanoparticles on the synthesized support materials. TEM images of the electrocatalysts are shown in Figure 6. The images show that the Pt nanoparticles are spherical and homogeneously distributed on the surface of the support materials. Particle size calculations were made using the software ImageJ from the images obtained from the TEM analysis; the results ranged from 3.76 to 3.49 nm for physically mixed support materials, which was close to those calculated with XRD. Hydrothermally synthesized electrocatalysts showed higher particle size values between 5.90 and 6.10 nm. Particle size calculations were also made using the Scherrer equation and the data obtained from XRD analysis. The data obtained from both analyses are presented in Table 3.

**Figure 7:**
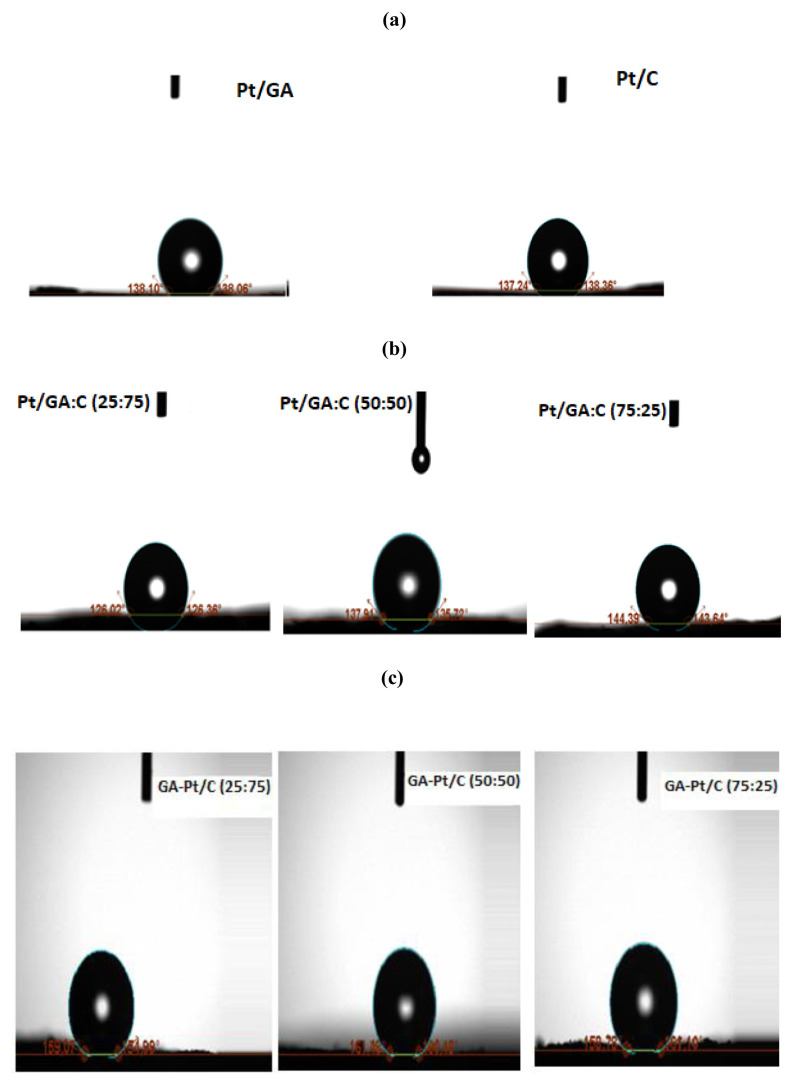
Contact angles of the GDEs prepared with the synthesized materials a) Pt/GA and Pt/CB, b) physically mixed GA:CB-supported Pt electrocatalysts, c) hydrothermally synthesized GA-Pt/C electrocatalysts.

One of the most significant factors in cell performance in PEM fuel cells is water management. Optimum membrane moisturization and the prevention of water flooding at the cathode can be achieved through optimal water management. To prevent water flooding at the cathode, water must be removed. If excess water is not effectively removed from the surface, the channels on the bipolar layer become clogged, and the gas sent to the system cannot effectively reach the catalyst surface. The use of hydrophobic materials in the catalyst structure is an effective method for controlling this situation [43]. Contact angle measurements of the gas diffusion GDEs prepared with the synthesized electrocatalysts are given in Figure 7. In these measurements, the degree of hydrophobicity of the electrode surface was evaluated. The average contact angle values for the GDEs are presented in Table 4. In physically mixed support materials, all electrode surfaces were hydrophobic, with the highest hydrophobicity being obtained on the electrode surface prepared with GA:CB (75:25)-supported Pt. In the hydrothermally synthesized electrocatalysts, all the surfaces were superhydrophobic.

### 3.2. PEM fuel cell test results

**Figure 8:**
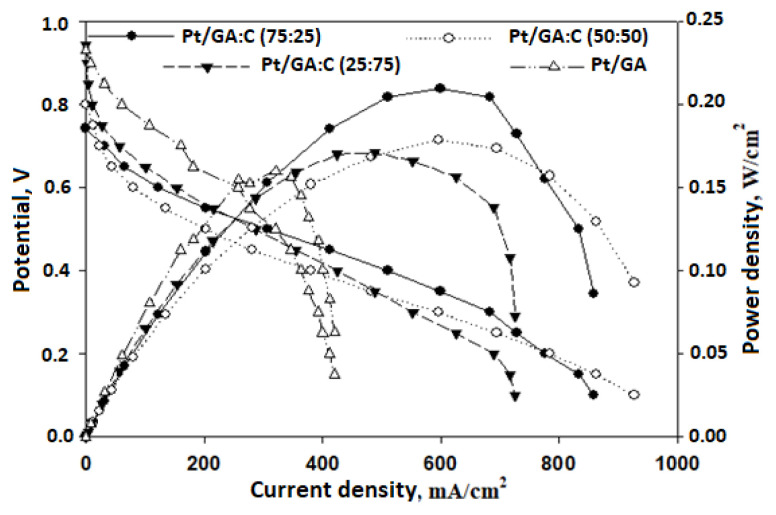
Polarization curves of GA and physically mixed GA:CB-supported Pt electrocatalysts.

The prepared structures were constructed to contain 0.4 mgPt/cm^2^ and the resulting polarization curves for Pt/GA and physically mixed electrocatalysts are shown in Figure 8. The highest current density and power density were 257.8 mA/cm^2^ and 0.15 W/cm^2^, respectively, at 0.6 V for the Pt/GA sample. However, a sharp decrease was observed in the polarization curve of the electrode prepared with the Pt/GA catalyst after 0.6 V. This decrease is thought to have been due to the high relative humidity within the cell [44]. Although the support material used is hydrophobic, water flooding in PEM fuel cells cannot be controlled solely by this parameter, and this is thought to be a factor in the performance degradation of Pt/GA. Excess moisture within the cell increased the intracellular resistance, limiting the reactions [45]. Here many parameters such as the physical characteristics of the components used and cell operating conditions (humidification amount, operating temperature, etc.) are influential [46]. At higher current densities, Pt/GA:CB (75:25%) gave a better performance when compared to the other electrocatalysts.

**Figure 9:**
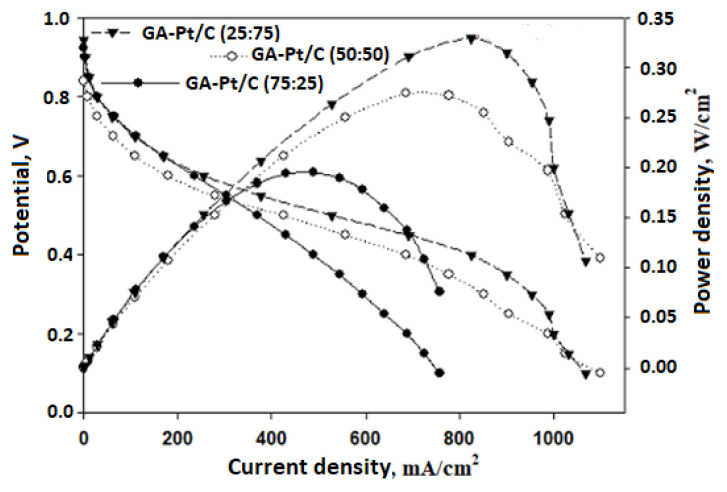
Polarization curves of hydrothermally synthesized GA-Pt/CB electrocatalysts.

The polarization curves of the GDEs prepared with the hydrothermally synthesized electrocatalysts are given in Figure 9. The highest current density was 257.8 mA/cm^2^ and the power density was 0.15 W/cm^2^ at 0.6 V for the Pt/GA sample (Figure 8). After Pt/GA, the best fuel cell performance belonged to the electrode prepared with the GA-Pt/CB (25:75) electrocatalyst, and a current density of 235.6 mA/cm^2^ and a power density of 0.196 W/cm^2^ at 0.6 V were obtained. Similar to the physically mixed electrocatalysts, at higher current densities the best performance was obtained for the GA-Pt/CB (25:75) electrocatalyst. All the electrocatalysts synthesized either physically or hydrothermally showed lower PEM fuel cell performance when compared to the commercial Pt/CB electrocatalyst (Tanaka, 67%) [47].

## 4. Conclusions

In the present study, GA-based Pt electrocatalysts were synthesized using two different synthesis routes. In the first route, electrocatalysts were synthesized over physically mixed GA:CB support materials. In the second route, GA was added hydrothermally to synthesized Pt/CB electrocatalysts. Following different synthesis routes resulted in different physical properties. The ID/IG ratios of the physically mixed support materials did not change with the addition of CB to GA, but the addition of Pt increased the defect sites in the structure. The ID/IG ratio increased as the amount of GA in the structure increased for the hydrothermally synthesized electrocatalysts. The BET surface areas of the physically mixed hybrid materials decreased with the addition of CB to GA. The hydrothermally synthesized electrocatalysts showed higher particle sizes when compared to the physically mixed ones. In the physically mixed support materials, all electrode surfaces were hydrophobic, with the highest hydrophobicity being obtained on the electrode surface prepared with GA:CB (75:25)-supported Pt. In the hydrothermally synthesized electrocatalysts all the surfaces were superhydrophobic. The hydrothermally synthesized electrocatalysts gave better PEM fuel cell performance when compared to the physically mixed ones.

## Figures and Tables

**Table 1:** Pt mass percentages of the synthesized electrocatalysts.

Materials	Pt (%)
**Physically mixed GA:C-supported Pt electrocatalysts**	

Pt/GA	14.5
Pt/GA:C (25:75)	23.6
Pt/GA:C (50:50)	11.6
Pt/GA:C (75:25)	13.4

**Hydrothermally synthesized GA-Pt/C electrocatalysts**	

GA-Pt/C (25:75)	11.3
GA-Pt/C (50:50)	11.1
GA-Pt/C (75:25)	12.8

**Table 2:** Structural properties of GA and physically mixed GA:CB support materials.

Support	BET surface area (m^2^/g)	Average pore diameter (nm)	BJH pore volume (cm^3^/g)
GA	265.3	3.9	0.2
GA:C (75:25)	228.7	4.1	0.3
GA:C (50:50)	186.9	4.9	0.4
GA:C (25:75)	178.6	5.6	0.4

**Table 3:** Particle sizes obtained from XRD and TEM.

Materials	Particle sizes from XRD (nm)	Particle sizes from TEM (nm)
**Physically mixed GA:C-supported Pt electrocatalysts**		

Pt/GA	3.60	3.76
Pt/GA:C (25:75)	3.48	3.57
Pt/GA:C (50:50)	3.46	3.50
Pt/GA:C (75:25)	3.23	3.49

**Hydrothermally synthesized GA-Pt/C electrocatalysts**		

GA-Pt/C (25:75)	6.30	6.10
GA-Pt/C (50:50)	6.10	5.90
GA-Pt/C (75:25)	5.90	5.90

**Table 4:** Contact angle values of the GDEs prepared with the Pt/GA, physically mixed Pt/GA:CB and hydrothermally synthesized GA-Pt/CB electrocatalysts.

Materials	Average contact angle (25 °C)
Pt/C	137.8

**GDEs of physically mixed GA:C-supported Pt electrocatalysts**	

Pt/GA	138.1
Pt/GA:C (75:25)	144.0
Pt/GA:C (50:50)	136.8
Pt/GA:C (25:75)	126.2

**GDEs of hydrothermally synthesized GA-Pt/C electrocatalysts**	

GA-Pt/C (25:75)	157.0
GA-Pt/C (50:50)	160.9
GA-Pt/C (75:25)	159.9
